# Tuberculinum

**Published:** 1894-01

**Authors:** Horace P. Holmes

**Affiliations:** Omaha, Neb.


### TUBERCULINUM.

Horace P. Holmes, M. D., Omaha, Neb.

Read before the International Hahnemannian Association June meeting, 1893.

Tuberculinum is one of those remedies which has come up to us along with the outgrowth of that peculiar system of therapeutics known as Isopathy. Where the isopathic idea originated is impossible to trace. Paracelsus wrote in such a way as to give little room for doubt that he believed in the occasional application of the theory that "the same cures the same." Instances are not wanting to trace a complete chain of evidence of the occasional use of isopathic remedies from the time of Paracelsus, early in the sixteenth century, down to the present day.

In regard to the particular isopathic remedy here chosen for a subject—Tuberculinum—we have many references, and more history than would be supposed at first thought. If one were to ask the great world who was first to use the diseased products of consumption as a cure for that malady, the universal answer would be—Koch. And yet we have records going back two hundred and fifty years, when an English physician, Dr. Robert Fludd, wrote: "*Sputum rejectum a pulmonico post debitam præparationem curat phthisin.*" Here Koch with his theory was antedated two hundred and fifty years. But as far as we are concerned, and for all practical purposes, we need to go back but sixty years to the time when our own Constantine Hering wrote, in 1833, that of the many remedies which would cure isopathically, he would advise "phthisine for phthisis." Here again Koch was antedated sixty years. The man, however, who has done more to put Tuberculinum, as a remedy, in the hands of his professional brethren, and who, so far as the writer can ascertain, was the first to actually prepare and use it for tuberculous troubles, and to have his work reported in regular publications, was Samuel Swan, M. D., of New York city. We should give all "honor to whom honor is due," and detract nothing from the helpful hints of those who have here and there thrown out suggestions which have been as rays of light to the followers. Hering suggested, Swan acted. Swan's work once established, Dr. Burnett wrote his excellent monograph on the *New Cure of Consumption*. Dr. Swan was some twenty years ahead of Koch, and when the latter famous scientist was paralyzing the world with the brilliancy of his supposed discovery, J. Compton Burnett, M. D., was writing his *Five Years' Experience in the New Cure of Consumption by Its Own Virus*. In our homœopathic literature we have a report of a case in the July, 1879, number of *The Organon*, a journal which was published for a short time in England. In the number referred to, Dr. Samuel Swan gives an exhaustive report of a complicated case where he deemed tuberculous influences were at work in his patient. The record says he prescribed Tuberculinum on the 18th day of November, 1877, and repeated the doses once a week until four doses in all were given. The permanent, curative results were something wonderful. The above article bears the date of January 17th, 1879, and, as stated, appeared in the July issue of the journal the same year. In the same volume is a second article on Tuberculinum by Dr. J. A. Biegler, of Rochester, N. Y., in which that gentleman reports the cure of a case of tubercular meningitis. The diagnosis had been confirmed by an eminent old school physician, so there can be no controversy on the point of diagnosis. Tuberculinum was administered on November 2d, 1878, and repeated at irregular intervals. The improvement was prompt and positive, and the cure complete and permanent.

The two principal preparations of Tuberculinum are Swan's and Burnett's. The latter is prepared by Heath and is sold under the name "Bacillinum Heathii." Dr. Swan made his Tuberculinum from the rich creamy pus, the contents of a freshly ruptured vomica from the lungs of a man in the last stages of phthisis tuberculosis. It was a patient of Dr. A. W. Pierson, of New York city, and the pus was potentized early in the '70s. A record of its use as early as 1874 is in existence. Dr. Burnett draws a comparison between the two preparations which seems ludicrous. He says of Swan's Tuberculinum, "The mode of obtaining it I felt to be too nasty."

His own æsthetic preparation was from a portion of a lung taken from an individual who had died from genuine tuberculosis. This post-mortem specimen, of course, contained all the morbid products of such a diseased condition—"bacilli, *dêbris*, ptomaines, and tubercles in all stages," and Dr. Burnett naïvely adds: "There is, moreover, nothing disgusting in this, which can hardly be said of sputal tuberculinum—one instinctively shrinks from it." Well, this is a matter of taste, and the writer is unable to draw a line as to the palatability or æsthetic position of one remedy over the other.

Dr. Burnett's change of name from the Tuberculinum of

Swan to Bacillinum is one open to serious criticism. It is not only unfair to the man who has done so much to bring the remedy into general use, and to whom Dr. Burnett is indebted for what little he first knew of Tuberculinum, but it is scientifically incorrect, so far as a specific name is concerned. Bacillinum is a term which might with equal propriety be applied to any cultures of bacilli or any morbid product containing them. It in no way specifies the one applicable to tuberculosis, and might with equal propriety apply to the diseased products of septicæmia, typhoid fever, cholera, glanders, leprosy, syphilis, malaria, and many other diseases in which specific bacilli are found. To be correct it should particularize the disease from which it came, and this Tuberculinum does. Moreover, Tuberculinum had been adopted and in use for more than sixteen years before Dr. Burnett wrote his first little book on the subject, and in which he took the liberty of changing the name Another error lies in the implied assertion that as bacilli were not found in Swan's first preparation they were probably not there, and as they were found in Burnett's material, it must follow that his was the more reliable of the two. It may seem unfortunate that Dr. Swan prepared his Tuberculinum and verified its efficacy some ten years before Koch discovered the bacillus tuberculosis. But such was the case. Had Dr. Swan waited until his work was coeval with Dr. Burnett's the bacillus would undoubtedly have been easily found in his first source of Tuberculinum. But as all the microbian scientists to-day agree that tubercle bacilli are found in the expectoration of typical cases of tuberculosis, and as that was the source of Swan's Tuberculinum, it is idle talk to intimate that the preparation is not so reliable as the one in which bacilli were found. It is an evident effort to appropriate the honor which belongs to another, and to detract from Dr. Swan's work the merit which is due him.

Koch's Lymph is prepared from the tuberculous processes in such a manner as to make it of little use to the homœopathic profession. The principal objection is that it is a compound, and not only that, but the chances are exceedingly probable that its composition may vary in character. In the first place, it is derived by artifically cultivating the tubercle bacilli in a suitable medium in order to obtain a quantity of the bacilli and their products. This culture fluid with its contents is filtered through procelain and then heated to a baking temperature. To this is added enough carbolic acid to thoroughly obliterate the germ action and it is then mixed with glycerine. Strange as it may seem, this preparation was found to have a most virulent action when diluted one thousand times, or to the third decimal attenuation. Whether the supposed curative action was due to the dynamic influences of the bacillic culture or to the action of the carbolic acid and glycerine, similar to the phenic acid preparations with which Dr. Declat thought to revolutionize thereapeutics, still remains to be settled. The remedy seemed to alleviate a few, killed a great many, and proved non-curative in almost every instance in which it was used according to Koch's instructions. But when Koch's Lymph fell into the hands of homœopathic practitioners and was diluted to the 6th or 10th decimal attenuations a curative action was developed which already promises wonderful results. Not only have the tuberculous lung affections been brought under a very decided control, but in other maladies as well the action of the remedy has proved its value. Dr. Marc Jousset, of Paris, has cured acute parenchymatous nephritis where there was one grain of albumen per litre, the albumen disappearing in a very few days. In experiments made on animals, it has been proven that Koch's Lymph produces acute parenchymatous nephritis, and hence the curative action of this remedy in Bright's disease and its homœopathicity to it.

The field of action to which Tuberculinum is applicable presents a wide range, not only for tuberculous affections of the lungs, but for all maladies which owe their origin or their chronicity to a tuberculous taint. Since the researches of those scientists who have devoted so much study to the microbian theory of diseases has incontestably proved that the so-called scrofulous affections of the glands known as chronic adenitis are, as a rule, but a latent tuberculosis, we are entitled to carry the reasoning a step farther, and claim that other inveterate chronic troubles, such as skin diseases, kidney difficulties, nervous affections, etc., are often but other forms of latent tuberculosis. It must lead us into a very close relationship with the psoric theory of Hahnemann and cause us to make a new differentiation. If psora is to be a name which expresses in a broad sense a dyscrasia which gives diseases a chronic foothold, then we must subdivide psora into different families of which tuberculosis will be one. Others will be found to have for their origin a syphilitic, gonorrhœic, or some other taint for which psora will be too indefinite a term, and for which a closer differentiation is necessary to diagnosis and prescribing. What Psorinum has done in its broad field of usefulness, Tuberculinum must do in those particular conditions where tuberculosis is the fundamental feature in the malady.

Tuberculinum will be found useful in either simple or more serious colds where there is a tendency to cause bronchitis. It is especially serviceable to those individuals who take cold very easily, and where the difficulty at once locates itself on the bronchial mucous membrane, causing a teasing, troublesome cough, which is slow to recover, under either time or the ordinary methods of treatment. For incipient tuberculosis, and also where a case is slowly but surely dragging a patient on to a serious tuberculous condition, this remedy has proved itself to be one of most excellent merit. For well developed cases of phthisis tuberculosis it is too much to expect that this, or any other remedy will cure in very many instances. But it already promises to be more efficacious than any other remedy. Based upon its indications, through provings of Koch's Lymph, it has promptly relieved acute parenchymatous nephritis, as has already been noted, and removed the albumen from the urine in from two to four days. May this not be a reasonable ground for inferring that Bright's disease is an affection really based on tuberculous tendencies? Tubercle bacilli are often found in the urine, and it is not at all improbable that affections, which many physicians pronounce Bright's disease, are in reality tuberculous conditions of the kidneys. A closer differentiation in these cases will be necessary in order to settle upon the etiology, as well as treatment.

Tubercular meningitis was one of the first diseases successfully treated with Tuberculinum, and analogous reasoning shows it indicated in tuberculous affections of the bowels. Tabes mesenterica and cholera infantum have been cured by this remedy. Tubercular affections of the bones and joints have been promptly benefited by this remedy, and Burnett's experience with ringworm proves its efficacy in skin diseases, and also shows that skin diseases may often be based on a tuberculous origin. Cases which have not recovered from la grippe, and which date their ill-health to the epidemic invasion of that malady, are as a rule, favorably influenced by Tuberculinum. Idiocy and cretinism, undoubtedly based on tuberculosis, have shown wonderful improvement when treated with Tuberculinum. Rheumatism and gout, chronic headaches, sleeplessness, general decline, accompanied by amenorrhœa in young girls, chronic diarrhœa, hæmorrhoids, chronic conjunctivitis, and granular ophthalmia, are among the many affections which have been benefited or cured with this remedy.

Provings of Tuberculinum may be found in Hering's *Guiding Symptoms*, Volume X ; in Allen's *Therapeutics to Gregg's Consumption* ; in different numbers of the *Homœopathic World* for 1891, provings of Koch's Lymph were published, and in the November number of the *Homœopathic Recorder*, is a proving of Bacillinum Heathii by R. Bocock, M. D. The first proving was made by Dr. Swan, and is thus far the principal one at our command. In all probability other provings will rapidly be made, and their additions joined to what we already have, making Tuberculinum one of the best proved remedies in our materia medica.

This remedy seems better adapted to blondes than to brunettes ; to the thin, slender individuals rather than the fleshy ; and to the mentally active, rather than those of sluggish dispositions. It is allied to Sulphur, Psorinum, and Carbo-veg., in being useful where the indicated remedy fails to act. It has the power of reviving the vital force, so that indicated remedies may regain an action, and good authority says an occasional dose of Tuberculinum is not interfered with by the intercurrent use of other remedies.

As to dosage, we can only advise the higher potencies. Koch killed patients with the 3d decimal attenuation, and all the successful results have been accomplished in our school with the 30th and higher potencies. Burnett favors the 100th and 200th, and all are emphatic in advising doses to be infrequently given at not shorter intervals than one week. In the writer's hands the most efficacious potencies, after months of experience with different potencies of Bacillinum Heathii, have been Swan's highest. In many instances in the writer's experience hæmoptysis, with sharp lancinating pains in the lungs have followed the administration of a simple dose of Swan's Tuberculinum—his highest potency. This has been too often verified to admit of doubt as to its being an aggravation caused by the remedy.

In closing, a word of caution will be *apropos*. We have in Tuberculinum a most valuable remedy, and at the same time capable of doing a vast amount of damage. The greatest care and judgment should be used in administering it, and the advice of those who have brought this remedy into prominence—to use only high potencies, and at the intervals of not less than one week—should not be ignored. There are already many physicians who will not use it because they are unable to get it in the 3d or 6th attenuation, and do not believe in a higher potency of any remedy. To such physicians it is imperative to say: Let this remedy alone until you can take the advice of those who know what they are talking about.

